# Age-related positivity effect on behavioural responses of dogs to human vocalisations

**DOI:** 10.1038/s41598-019-56636-z

**Published:** 2019-12-27

**Authors:** Iris Smit, Dora Szabo, Enikő Kubinyi

**Affiliations:** 1 Department of Ethology, Eötvös Loránd University, Budapest, 1117 Hungary; 2 HAS University of Applied Sciences, ‘s-Hertogenbosch, 5223DE The Netherlands

**Keywords:** Animal behaviour, Cognitive ageing

## Abstract

Age-related changes in the brain can alter how emotions are processed. In humans, valence specific changes in attention and memory were reported with increasing age, i.e. older people are less attentive toward and experience fewer negative emotions, while processing of positive emotions remains intact. Little is yet known about this “positivity effect” in non-human animals. We tested young (n = 21, 1–5 years) and old (n = 19, >10 years) family dogs with positive (laugh), negative (cry), and neutral (hiccup, cough) human vocalisations and investigated age-related differences in their behavioural reactions. Only dogs with intact hearing were analysed and the selected sound samples were balanced regarding mean and fundamental frequencies between valence categories. Compared to young dogs, old individuals reacted slower only to the negative sounds and there was no significant difference in the duration of the reactions between groups. The selective response of the aged dogs to the sound stimuli suggests that the results cannot be explained by general cognitive and/or perceptual decline. and supports the presence of an age-related positivity effect in dogs, too. Similarities in emotional processing between humans and dogs may imply analogous changes in subcortical emotional processing in the canine brain during ageing.

## Introduction

Changes in the brain during ageing influence how adults process emotions^1^. Whereas the processing of positive emotions remains more or less stable, older adults experience fewer negative emotions. This phenomenon, referred to as age-related positivity effect, is present both in attention and memory^2^. For example, Mather & Carstensen^3^ found that older adults spent less time looking at negative features of portraits than on positive features, and that the time spent looking at negative features was shorter than in younger adults. This form of selective attention has an effect on memory, because higher attention to positive features can lead to stronger memories^4^.

The positivity effect was also observed in the auditory domain, in regard to processing emotional prosody and valence. Prosody is defined as the properties of larger units of vocalisations that can indicate the emotional state of a speaker^5^, and valence describes the attractiveness/averseness or emotional association of a stimulus^6^. Older adults have more trouble identifying emotional valence in sounds and pitch perception of emotional prosody can be impaired as well^7,8^. A cross-sectional study on the perception of both sounds and faces with different emotional valences showed that aged adults have a decreased ability to recognise both sadness and anger, whereas the recognition of other emotions remained intact^9^.

As in humans, the functions of the canine brain are known to decline with age^10^. Both the cognitive functions of the medial temporal lobe and the prefrontal cortex are impaired in old dogs, as demonstrated in an indirect behavioural study by Head *et al.*^11^. In this study, older dogs performed worse on several cognitive behaviour tests that were used to assess age-dependent cognitive decline. This was later supported by several other behavioural studies^12–15^. An MRI study by Tapp *et al.*^16^. confirmed that in 8–15 year old beagles the frontal lobe and the hippocampal volumes declined with age. Similar ageing effects have been found in other animal models, including mice^17^, rats^18^, cats^19^ and primates^20^. However, while age-effects on responsiveness in regard to certain sound characteristics like frequency have been studied in animal models^21^, age-effects in the processing of emotionally loaded stimuli have not been studied in non-human animals yet. Information will add to our knowledge about the evolution of emotion-processing and the validity of animal species as aging models.

Family dogs are able to differentiate between human emotions. For instance, dogs were able to discriminate between happy and blank faces on photographs^22^ and they were most reactive to commands when their owners displayed happiness instead of neutrality or disgust with body language and sound^23^. A cross-modal, preferential looking paradigm study showed that dogs spontaneously paired positive and negative emotions of both human and conspecific faces on pictures with pre-recorded emotional sounds^24^. Emotion processing was also tested in the head turning paradigm, where dogs reacted differently towards positive and negative sounds. This supports that dogs spontaneously discriminate between positive and negative human emotions. In addition to discriminating between the emotions, research on emotional contagion has shown that dogs are affected by both visual and auditory emotional expressions. Dogs licked their mouth more frequently when they faced pictures of human and dog faces with negative emotional expression, regardless the valence of the sound presented at the same time^25^. Dogs showed significantly more arousal and stress related behaviours following negative emotional sounds compared to positive and non-emotional sounds they were exposed to, suggesting they matched the emotional valence in the negative sounds^26^. Using a non-invasive functional magnetic resonance (fMRI) procedure with awake dogs and humans, Andics *et al*.^27^ showed the existence of specific voice areas and the presence of emotional valence sensitivity in dogs’ brains.

We hypothesised that dogs show age-related positivity effect similar to humans, as both the general age-related changes in the brain, and the brain regions involved in auditory processing are similar in humans and dogs. Specifically, the present study focused on whether there are age-related differences in the spontaneous behavioural reaction of dogs to human vocalisations with positive, negative, and neutral emotional valences during a sound playback test. We decided to use human vocalisations because (1) we have less information regarding the perceived valence of dog vocalisations by other dogs and (2) not all type of dog vocalisations are directed to other dogs, some vocalisations have been showed to be human directed^28^. This could lead to unexpected variations in the interpretation of the sounds by the dogs. (3) The huge variance in body size among dogs has the potential to introduce additional confounding factors (e.g. a small dog or a dog with negative previous experiences may perceive certain dog vocalisations as more threatening, while from some large dogs small dog vocalizations may elicit predatory responses)^29^, which we wanted to exclude. We compared latency to react and the latency of recovery of aged and young dogs with intact hearing. Via this setup we investigated whether aged dogs were (1) less responsive to all sounds in general due to e.g. general cognitive decline or (2) selectively less responsive toward negative stimuli than young dogs corresponding to a positivity effect.

## Methods

### Ethics statement

The behavioural observations conducted in this study were not identified as animal experiments by the Hungarian Animal Protection Act (“1998. évi XXVIII. Törvény”, 3. §/9.), which identifies animal experiments, as this study was non-invasive. The application number of the ethical commission for studies performed by the Senior Family Dog Project is PE/EA/2019-5/2017. Each owner filled in a consent form stating that they have been informed of the tests.

### Subjects

A total of 46 family dogs were tested, of which six had to be excluded from the analysis. Reasons for exclusion included excessive stress reactions to the sounds (n = 1), hearing impairments (n = 1), owner interference during the sound playback (n = 1) and technical problems (n = 3). Finally, n = 21 young dogs (1–5 years) and n = 19 old (>10 years) dogs were analysed in the study. Only dogs between 4 and 30 kg took part in the study, with a mean weight of 17 for old dogs and 16 for young dogs. This restriction was implemented because dogs with different sizes could exhibit a difference in aging rate. The female to male ratio was 2:5 (6:15 female to male for the young age group and 6:13 for the old age group). The dogs were required to be experimentally naive for sound playback studies. The hearing of the subjects was assessed by calling the dogs’ name and via using a rattler behind them, by both the owner and the experimenter in a test following the experiment. Dogs which showed any signs of hearing impairments were excluded from the analysis (n = 1). Owners were recruited via online promotion and from the Department of Ethology’s database.

#### Test room

**Figure 1:**
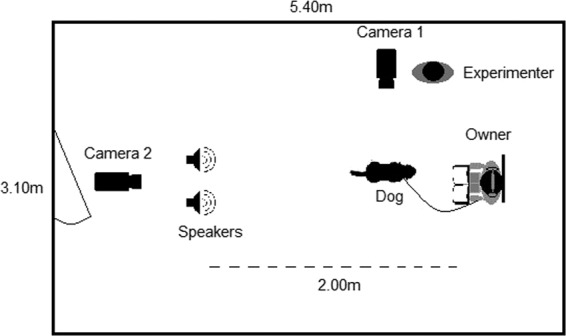
Test room set-up. The owner was positioned in the back of the room and the leashed dog was laying down in front of them. The experimenter was standing next to camera 1, 1.25 meters away from the owner and dog. Camera 2 was positioned behind the speakers, which were positioned 2 meters in front of the owner. The owner was reading a magazine and listening to music via headphones to minimise their effect on the dog’s reaction.

The tests were conducted at a room of the Department of Ethology. The room measured 3.10 × 5.40 metres. The owner was positioned on a chair in the back of the room (1 metre from the front wall) facing the speaker set (2× Logitech S-02648, 230 V ~50 Hz 40 mA), which were placed together 2 metres in front of the chair with a 38 cm distance between the two speakers (Fig. 1). The experimenter was standing next to camera 1, positioned next to the wall 1.25 metres away from the chair. Camera 2 was positioned 80 centimetres behind the speakers. The speakers were connected to a laptop and a media pointer was used to change the sounds using PowerPoint. The dog was positioned in front of the owner, with its back to the speakers, facing either the owner or the side walls.

#### Stimuli

The 6 stimuli used (2 positive, 2 negative, 2 neutral, all non-linguistic) were pre-selected from published studies. The positive and negative sounds were chosen from a study by Anikin & Persson^30^ that validated a corpus of 260 human vocalisations using a survey on 90 participants with different nationalities who rated the sounds. They were non-acted sounds taken from real life video clips. The negative sounds featured a cry by a male and a female and the positive sounds were a male and a female laugh. As this corpus lacked neutral vocalisations, the two neutral stimuli, a hiccup and a cough, were sound effects taken from Youtube. The cough was male and the gender of the hiccup sound was not identified in the description of the source video. All stimuli had a duration of 5 seconds, were recorded in mono and had a RMS volume of 20 dB, limited on −3.5 dB with a soft limiter. The sounds were edited using Audacity 2.2.2. Further details of the sounds can be found in Table 1.

**Table 1:** Emotional stimuli used and their sound ID, corresponding emotion, speaker gender, Root Mean Square volume, Mean and Fundamental frequency and Mean of the recorded play level.

Sound ID	Emotion	Speaker gender	RMS volume	Mean frequency	Fundamental Frequency	Mean recorded play level
neuSTIM1	Neutral	Unknown	20 dB	633 Hz	325 Hz	56.26 dB
neuSTIM2	Neutral	Male	20 dB	500 Hz	329 Hz	70.93 dB
posSTIM1	Positive	Female	20 dB	1040 Hz	128 Hz	74.37 dB
posSTIM2	Positive	Male	20 dB	1828 Hz	318 Hz	75.95 dB
negSTIM1	Negative	Male	20 dB	1481 Hz	350 Hz	69.38 dB
negSTIM2	Negative	Female	20 dB	1465 Hz	155 Hz	73.50 dB

**Figure 2:**
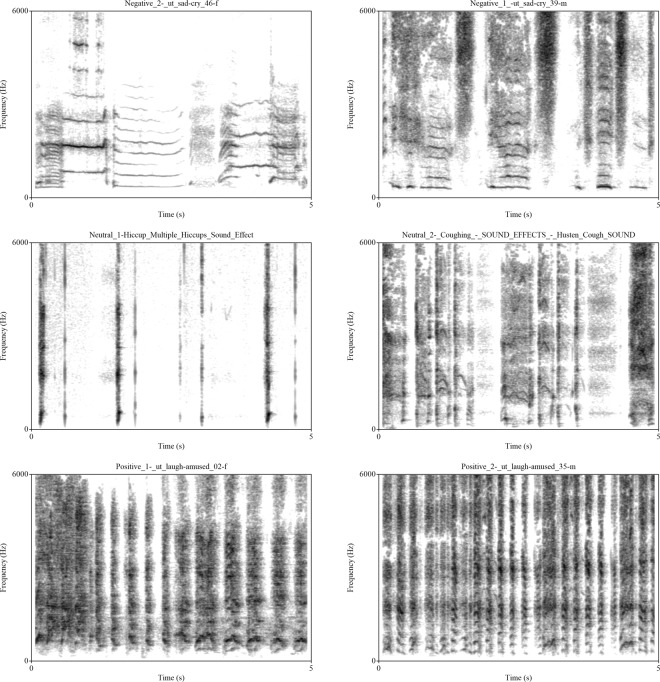
Sonogram of the used stimuli. Made with Praat. Showing 0–6000 Hz with a 0.05 s timewindow and 60 dB dynamic range. The two negative stimuli are displayed at the top, followed by the two neutral sounds in the middle row and the two positive sounds at the bottom.

Logarithmic power spectral density was calculated using the Long Term Average Spectrum (LTAS) function in^31^, with a 100 Hz bandwidth between bands of 0–1000 and 1000–4000. For the resulting values see Table 2. Sonograms of the sounds (Fig. 2), with a range of 0–6000 Hz and a 0.05 s time window and 60 dB dynamic range, were created using Praat.

**Table 2:** Logarithmic power spectral density of the stimuli. Calculated by the LTAS function on Praat with 100 Hz bandwidth. Showing the Mean, SD and Slope between bands of 0–1000 and 1000–4000 Hz. All values are shown in dB and are expressed relative to 2 × 10^−5^ Pa.

Sound ID	Mean	SD	Slope
neuSTIM1	−8.0536	19.0972	−9.3396
neuSTIM2	6.3948	24.8805	−5.8144
posSTIM1	−9.0254	27.5312	−1.5017
posSTIM2	4.3246	24.3179	0.8125
negSTIM1	9.7657	21.2974	1.1894
negSTIM2	−7.7492	18.8035	−10.3067

The play level of the sounds was measured using a Voltcraft SL-200 Digital Sound Level Meter, 2 meters from the speakers. The mean level of the sounds during the experiment was 70.08 dB with a reference level of (x microPa) and the background sound level had an average of 39.2 dB.

#### Procedure

The owner was sitting on the chair reading a magazine and listening to music, therefore the test sounds were inaudible for him/her. The dog was given time to acclimatise and lie down on its own. If it did not lie down within a minute, or if it lied down facing the speakers, the owner was asked to position the dog in front of him/her so it was laying down with either its back or side to the speakers. Once the dog lied down and was looking away from the speaker, the sound playback started, and the first sound (testSTIM1) was played. Once the dog recovered from the sound, but at least 10 seconds later and maximum 1 minute later, the owner was asked to reposition the dog if necessary. Once the dog was again not focused on environmental stimuli (i.e. it was passive), the second sound (testSTIM2) was played. This was repeated for the third sound (testSTIM3). After a 5-minute break outside the test room in which the owner was free to interact with the dog, the trial was repeated with the 4^th^–6^th^ sounds (testSTIM4, 5 and 6). The playbacks of testSTIM1–6 were semi-randomised for each subject. Each session contained 1 positive, 1 negative and 1 neutral stimulus, therefore after the two sessions all subjects were exposed to two positive, two negative and two neutral stimuli. Video recording of the dogs’ behaviour happened continuously during the trials and the behaviours were analysed based on the videos.

#### Data collection

Latency to react. Time in milliseconds from stimulus onset till the dog started its head/ear turn towards the source of the sound. We stopped the observation after 10 seconds.

Latency to recover. Time in milliseconds from latency to react until the dog started to turn away from the source of the sound. We stopped the observation after 60 seconds.

One observer scored the latencies and behaviours blindly, without hearing the sounds during scoring, after which the same observer coded the latencies using the audio stream for determining stimulus onset, based on the previously coded reactions. The second observer scored 120 trials using the same method.

### Statistical analysis

R (version 3.5.1.) was used for the statistical analysis^32^. Inter-observer reliability analysis of the latencies was calculated via coding 25% of the data by a second, trained coder. Agreement was calculated using intraclass correlation coefficients (ICC) in a two-way mixed-effects model with a 95% confident interval on consistency in SPSS 22^33^. Normality of the latency to react and latency to recover was checked using Q-Q normality plots, which showed neither of the variables were normally distributed. Because of censoring within the reactivity and latency data (e.g. when the dog did not react within 10 seconds or when the dog did not redirect its attention within 60 seconds), Survival Analysis Methods were used.

First, to check whether there were any differences between the two samples within the categories we used Kaplan Meier estimates with the main factor of Sound ID and a post-hoc pair-wise comparison. Survival analysis was used because right censoring occurred during the tests. To compare the reaction and recover latencies of young and old dogs Kaplan Meier estimates were used, with the main factors being age group and sound category. Mixed Effects Cox Regression Models were used to analyse the effects of age group, emotion category and playback order on the latency to react and recover with subjects as random variable and the sound categories, trial order and the dogs’ age as factor. The young age group, the neutral sound category and trial 1 were set as reference categories. Cox proportional hazards were used to analyse the confidence intervals. Since there was a trial effect, the Cox models were also run on a dataset including only the first trial for each dog. The dogs that did not react were classified as censored within the latency to react model and not used in the latency to recover model. Dogs that did not recover were classified as censored in the latency to recover model in that specific trial. Exploratory analysis with a dredge function in R showed no suggested effects of sex, thus it was excluded from the models. The dredge function also showed improved models without interactions.

Reactivity and recovery were also tested with a binominal Generalized linear mixed model to compare the number of censored trials of the old and young dogs in the different sound categories. It also included the factors age group, sound category and trial, using the subject as random variable.

## Results

Intraclass correlation coefficients (ICC) for latency to react and latency to recover were excellent (0.924 and 0.793, respectively).

A log-rank test revealed no differences in the latencies to react and recover between the two different sounds within a category. Based on this, we analysed the reactions within a valence category (e.g. cry1 and cry2) together.

### Latency to react

Out of a total of 240 trials, there were 23 trials (from 15 dogs) in which the dog did not react within 10 seconds from the onset of the stimuli (Table 3). This resulted in a total of 117 trials with young dogs with a mean reaction time of 1231.43 msec (SD = 2631.10) and 100 trials with old dogs with a mean reaction time of 1686.84 msec, SE = 3179.12). A generalized linear mixed model with binomial distribution showed no significant effects of age group (β = −0.76, SE = 0.69, p = 0.27) or sound category (positive: (β = 0.14, SE = 0.63, p = 0.81), negative: (β = −0.07, SE = 0.60, p = 0.90)) on the dogs’ reactivity. However, a trial effect was found, dogs were less likely to react in Trial 6 than in Trial 1 (β = −2.23, SE = 0.91, p = 0.014).

**Table 3:** Number of censoring in the latency to react and recover. Showing the total censoring per age group and the number of censored trials in each sound category and their percentages of the total number of trials out of a total of 47 dogs and 240 trials.

Age group	Total n	No reaction within 10 sec				No recover within 60 sec			
		n	Positive	Negative	Neutral	n	Positive	Negative	Neutral
Young	126(100%)	9 (7%)	3(2%)	1(1%)	5(4%)	1(1%)	1(1%)	0	0
Old	114(100%)	14(12%)	4(4%)	7(6%)	3(3%)	7(6%)	0	5(4%)	2(2%)

#### Survival probability

**Figure 3:**
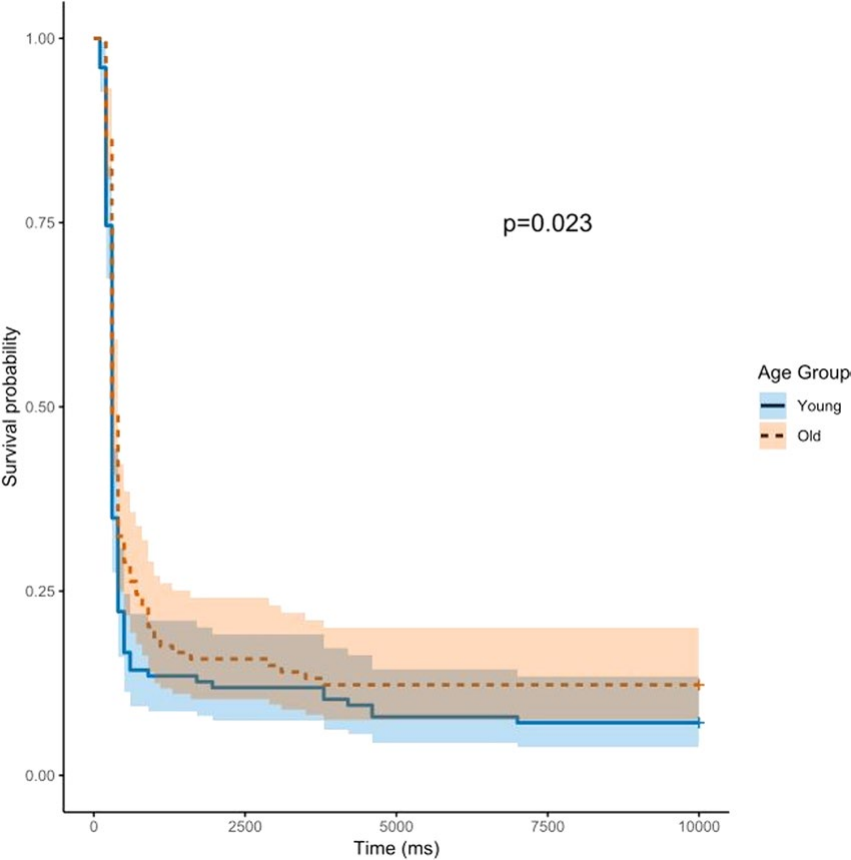
Kaplan Meier survival curve estimates of dogs’ latencies to react comparing trials with young dogs with trials with old dogs. The blue continuous line represents the young age category and the red interrupted line represents the old age category.

**Figure 4:**
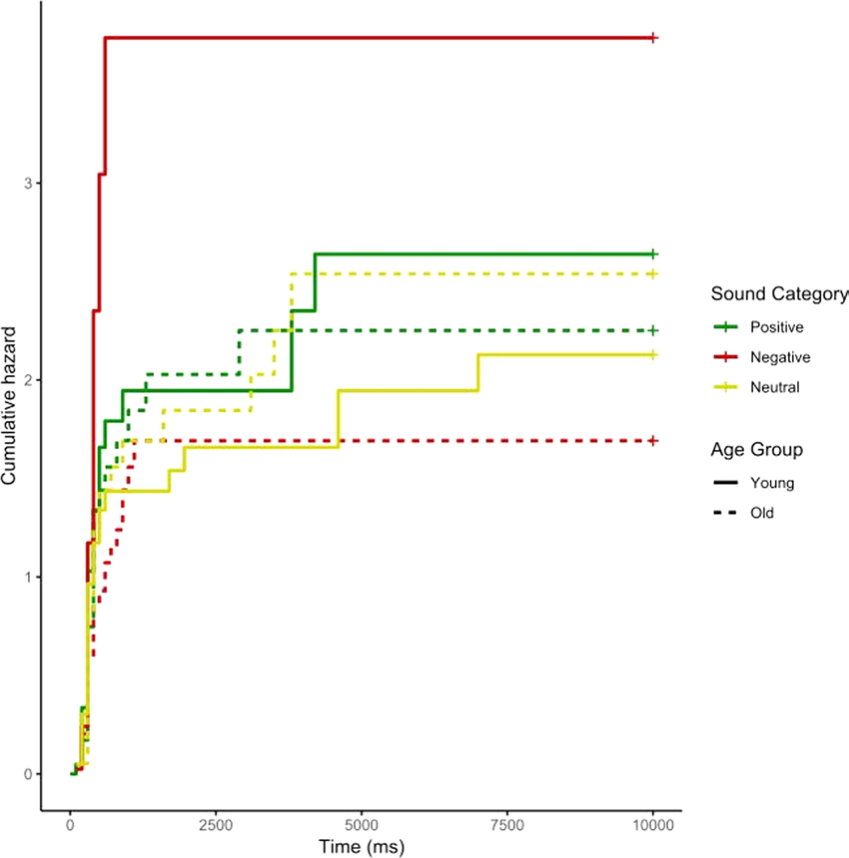
Cumulative hazards plot for the latency to react, grouped per age and sound category. The positive, neutral and negative sound categories are represented by the green, yellow and red coloured lines respectively. The young age group is shown with a solid line and the old age group with an interrupted line.

A log-rank test showed that old dogs responded to the sounds with a longer latency (Chi X^2^ = 5.2 p = 0.02) (Fig. 3). The difference between young and old dogs within the negative sound category was significant (Young: (95%CI: 300; 300), Old: (95%CI: 300; 800), p = 0.021). Within the negative sound category, young dogs showed an increased hazard to react compared to the old dogs (Fig. 4). We found no similar pattern within the neutral (Young: (95%CI: 300; 400), Old: (95%CI: 300; 400), p = 0.913) or the positive sound category (Young: (95%CI: 300; 400), Old: (95%CI: 300; 400), p = 0.521). This indicates that old dogs reacted significantly slower than young dogs only in the negative sound category.

#### Mixed effects cox regression

We tested whether dogs became habituated to the sounds across trials via a Mixed Effects Cox regression model. The cumulative hazard results of the Cox regression showed a significant hazard decrease (exp(β) = <1) in trial 5 and 6, of 0.56 (exp(β) = 0.57, 95%CI = (0.37;0.94), p = 0.03) and 1.04 (exp(β) = 0.35, 95%CI = (0.25;0.67), p < 0.001) respectively (for statistical details see Table 4). An exp(β) below 1 for these factors suggests an increased latency to react, i.e. dogs habituated to the sounds in trial 5 and 6.

**Table 4:** Cox Mixed Effects regression results for latency to react. Showing p-values, the coefficient (β) ± the standard error, hazard ratio (exp(β)) and the 95% confidence interval for a model including trial 1–6 and a model including only 1 trial. Trial 1, age group young and sound category neutral are set as reference categories. The significant values (p < 0.05) are represented in bold.

	Trial 1–6				Trial 1			
	p-val	β(SE)	exp(β)	95% CI	p-val	β(SE)	exp(β)	95% CI
Trial 2	0.65	0.11 ± 0.24	1.11	0.69;1.70	—	—	—	—
Trial 3	0.61	−0.13 ± 0.25	0.88	0.54;1.34	—	—	—	—
Trial 4	0.43	0.19 ± 0.24	1.21	0.71;1.77	—	—	—	—
Trial 5	0.024	−0.56 ± 0.25	0.57	0.37;0.94	—	—	—	—
Trial 6	0.0001	−1.04 ± 0.26	0.35	0.25;0.67	—	—	—	—
Age group old	0.084	−0.43 ± 0.25	0.64	0.52;0.89	0.0064	−1.11 ± 0.41	0.33	0.16;0.74
Sound category positive	0.40	0.14 ± 0.17	1.15	0.83;1.60	0.7600	0.13 ± 0.42	1.14	0.52;2.46
Sound category negative	0.56	0.10 ± 0.17	1.11	0.78;1.52	0.0380	−0.99 ± 0.48	0.37	0.16;0.93

### Latency to recover

The trials where the dogs did not react were excluded (due to the lack of latency to recover), resulting in a total of 217 trials being used for the analysis of the latency to recover. Out of a total of 217 trials in which the dog reacted, 5 dogs did not recover in 8 different trials (i.e. did not return to passive, unfocused state) within the time limit of 60 seconds after the onset of sound, or before another event, (e.g. due to owner interference or external distraction) (Table 3). The 116 trials with young dogs had a mean recovery time of of 10381.2 msec (SD = 7802.38) and old dogs had a mean of 10566 msec (SE = 11554.65).

#### Survival probability

The log-rank test showed that there was no significant difference in the latency to recover between young and old dogs (Young: (95% CI: 7700msec; 9800 msec), Old: (95% CI: 6100 msec; 9100 msec), p > 0.9). A Cumulative Hazard analysis paired with a log-rank test showed no age differences in recovery time from the negative (Young: (95%CI: 7500; 10700), Old: (95% CI: 7000; 13000), p = 0.93), positive (Young: (95% CI: 7300; 11300), Old: (95% CI :5500; 9100), p = 0.93) or neutral (Young: (95% CI: 7100; 11100), Old: (95% CI: 5600; 10800), p = 0.93) sounds. This indicates that there was no significant difference in latency to recover between young and old dogs in any sound category.

#### Mixed effects cox regression

The results of the Cox model (Cox mixed-effects model fit by maximum likelihood) showed a significant effect only in case of trial 4 (for statistical details see Table 5). The hazard of the latency to recover increased (exp(β) = > 1) in trial 4 by 0.19 (exp(β) = 2.25, 95% CI = (1.14;2.88), p = 0.001), showing that the dogs had a significantly shorter latency to recover during the first trial after the short break.

**Table 5:** Cox Mixed Effects results for latency to recover. Showing p-values, the coefficient (β) ± the standard error, hazard ratio (exp(β)) and the 95% confidence interval for a model including trial 1–6 and a model including only 1 trial. Trial 1, age group young and sound category neutral are set as reference categories. The significant p values (p < 0.05) are represented in bold.

	Trial 1–6				Trial 1			
	p-val	β(SE)	exp(β)	95% CI	p-val	β(SE)	exp(β)	95% CI
Trial 2	0.310	0.25 ± 0.25	1.29	0.72;1.80	—	—	—	—
Trial 3	0.700	0.10 ± 0.26	1.10	0.71;1.80	—	—	—	—
Trial 4	0.002	0.81 ± 0.26	2.25	1.14;2.88	—	—	—	—
Trial 5	0.090	0.44 ± 0.26	1.56	0.74;1.89	—	—	—	—
Trial 6	0.240	0.33 ± 0.28	1.39	0.68;1.85	—	—	—	—
Age group old	0.600	0.16 ± 0.32	1.18	0.78;1.35	0.80	0.09 ± 0.37	1.10	0.54;2.25
Sound category positive	0.240	0.21 ± 0.18	1.24	0.84;1.63	0.74	−0.14 ± 0.42	0.87	0.38;1.98
Sound category negative	0.280	−0.20 ± 0.19	0.82	0.67;1.33	0.42	0.35 ± 0.44	1.42	0.60;3.37

## Discussion

Cognitive decline in older dogs, which is thought to be linked to the temporal and frontal cortex^34^ can cause longer processing, namely slower reaction times and a longer latency to recover^11,16,35^. However, in the current study we showed that older dogs are not simply less responsive or slower to respond to the used stimuli in general. Latency to react did differ significantly between young and old dogs, but only in the negative sound category where the young dogs reacted faster than the old dogs, while latency to recover was not different between young and old dogs. The number of not recovering dogs also did not significantly differ between the old and young dogs across the different sound categories. Thus, it can be concluded that the old dogs that passed the hearing test were not less responsive to all sounds and the difference is more likely caused by changes in the processing of certain sound types than by general cognitive decline.

While attention span is generally decreased in older dogs^36^ and the likelihood to recover is smaller than in younger dogs^35^, we found no differences between old dogs and young dogs in whether they recovered from the stimuli within 60 seconds. Most dogs kept their attention towards the speakers after the sound with a duration of 5 seconds had ended, suggesting that the latency to recover in the current setup may not indicate attention to the sound per se but rather the behavioural changes following the arousal induced by it. Testing the phenomenon in a different setup, for example with projected images like in Racca *et al.*^37^ could provide information whether attention span toward emotional stimuli also changes in aging dogs. Alternatively, comparing the performance of young and old dogs trained to classify portraits based on the displayed emotions^38^ could reveal whether the positivity effects extends into the classification performance of old dogs and whether attention toward negative stimuli is selectively decreased in old dogs during an active choice task.

It is suggested that prosody discrimination in humans is, among other factors, is based on the duration and fundamental frequency of the sounds^39,40^. The effect of differences in prosody in dogs has been observed in regard to stress and arousal indicating behaviours^25,26^. The sounds used in this study were similar in their fundamental frequency (Table 1) and they all had a duration of 5 seconds. Additionally, in the selected sound set the negative samples did not deviate strongly from the other samples (the two negative samples were not the two most extreme ones neither regarding mean nor fundamental frequencies, see Table 1), meaning that the findings cannot be explained based on e.g. diminished reactivity to above/below a certain frequency range.

After multiple human studies reporting an age-related positivity effect^9,41,42^, the results of our study suggests the presence of a similar valence related phenomenon in dogs. While a previous study by Siniscalchi *et al*.^35^, in line with our current results showed a significant difference between young and old dogs in reactivity to emotional sounds in general, our study is the first to look at the role of valence in reactions of aging dogs and the presence of the positivity effect. Further studies including more emotional categories are needed to investigate the level of similarity between positivity effect in dogs and humans, for instance whether the processing of other negative emotional vocalisation (e.g. anger) are also affected in dogs, similarly to humans^9^.

Multiple theories have been proposed to explain the positivity effect in humans. The two main mechanisms differ in regard to their complexity, required level of abstract thought and the involved brain areas. The ageing-brain model ascribes changes in the processes in the anterior cingulate gyrus to downregulate the response of the amygdala to negative stimuli and thereby influence the way these emotions are processed and reacted upon^2,43^. Downregulating the amygdala has been connected to a higher level of emotional control and emotion regulation^44^. In contrast, the Emotional Selectivity Theory^3^ proposed the role of cognitive control in the shift in attention and memory away from negative towards more positive emotions. Based on the Emotional Selectivity Theory, these changes are caused by greater emotion regulation of older adults, and a change in perception of experiences, with positive experiences being valued higher by older individuals due to awareness of time left alive^3,43^. Since dogs are less likely to reflect upon their longevity and ultimate death and have yet to show signs of both emotion regulation and awareness of future life events, the current results cannot be explained by a positivity effect resulting from the Emotional Selectivity Theory. Thus, the presence of an age-related positivity effect in dogs supports the ageing-brain model.

Degeneration in the brain of older dogs is mainly found in the frontal and temporal cortex^34^. Processing of auditory stimuli is associated with the temporal lobe, while the attention to stimuli is regulated in the frontal lobe^45^. An effect of the similar degeneration in humans is presbycusis, the reduced ability to differentiate between acoustic properties and hearing loss. For example, older individuals have trouble identifying differences in sound duration, hearing short silent gaps in an auditory stimuli (temporal gap detection)^46^, hearing in noisy environments, speech processing^47^ and discrimination of complex stimuli^48^. Presbycusis is induced by cochlear degeneration, which causes hearing loss, primarily to the mid to high frequencies^49^. Loss of cochlear function has the same effect in dogs^50^. Ter Haar *et al*. found that hearing loss starts in dogs of around 8 years of age and are most prominent in frequencies ranging from 8–32 kHz^51^. In the current study, the negative sounds did not differ in frequency and average spectral density from the positive and neutral sounds, suggesting that presbycusis in the old dogs is not the cause of the differences in the latency to react.

There are multiple similarities in the processing of emotional human sounds in both the primary and secondary auditory regions between humans and dogs^27^. Processes and interactions involved in the prosody discrimination are also suggested to take place mainly in the frontal and temporal lobe^52–55^; where the anterior cingulate gyrus (in the primary auditory cortex of the temporal cortex) and amygdala (in the prefrontal cortex) are located. With age, the amygdala is known to decrease in volume^56^ and this is shown to affect the way emotional cues are processed in humans^57^.

There are two general pathways for auditory processing in the brain; the thalamic, or subcortical, and the cortical pathway^58,59^. It has been suggested that the subcortical pathway subconsciously processes sounds and visual stimuli before the cortical level, to provide the amygdala with a brief preliminary characterisation before further and slower processing using the cortical pathway takes place^59,60^. Auditory nerve fibres located in the brainstem are involved in the preliminary processing of fundamental frequency and harmonic cues in sounds^61^. Harmonic cues, like timbre, are used by the auditory regions in the brainstem to encode sounds that are not easily recognisable by frequency^62^. Auditory brainstem response (ABR) peaks are seen to be reduced in older adults, which suggests that the number and synchrony of the auditory nerve fibres are reduced^63^.

The fact that in our study the latency to react, but not the latency to recover showed significant differences between the old and young dogs in the negative sound category may suggest that the age-related changes take place in subcortical processing rather than in cortical processing, involving both the ageing of auditory nerve fibres of the brainstem and the degeneration of the amygdala. However, further research involving brain imaging is necessary to determine the specific cause of these effects, the brain regions involved and nature of the differences. Studying age-related differences in the processing of emotional stimuli in animals allows us to deepen our understanding regarding the positivity effect in different species and can give us more insight into the biological changes of the ageing brain, affecting how older individuals perceive and process their social environment.

## Supplementary information

Supplementary Information.

## Data Availability

All data generated during or analysed during the current study are included in this published article (and its Supplementary Information files). The supplementary dataset contains all measured variables.
